# angaGEDUCI: *Anopheles gambiae *gene expression database with integrated comparative algorithms for identifying conserved DNA motifs in promoter sequences

**DOI:** 10.1186/1471-2164-7-116

**Published:** 2006-05-17

**Authors:** Sumudu N Dissanayake, Osvaldo Marinotti, Jose Marcos C Ribeiro, Anthony A James

**Affiliations:** 1 Department of Molecular Biology and Biochemistry, University of California, Irvine, CA 92697, USA; 2 Laboratory of Malaria and Vector Research, National Institutes of Health (NIH/NIAID), Rockville, MD 20852, USA; 3 Department of Microbiology and Molecular Genetics, University of California, Irvine, CA 92697, USA

## Abstract

**Background:**

The completed sequence of the *Anopheles gambiae *genome has enabled genome-wide analyses of gene expression and regulation in this principal vector of human malaria. These investigations have created a demand for efficient methods of cataloguing and analyzing the large quantities of data that have been produced. The organization of genome-wide data into one unified database makes possible the efficient identification of spatial and temporal patterns of gene expression, and by pairing these findings with comparative algorithms, may offer a tool to gain insight into the molecular mechanisms that regulate these expression patterns.

**Description:**

We provide a publicly-accessible database and integrated data-mining tool, angaGEDUCI, that unifies 1) stage- and tissue-specific microarray analyses of gene expression in *An. gambiae *at different developmental stages and temporal separations following a bloodmeal, 2) functional gene annotation, 3) genomic sequence data, and 4) promoter sequence comparison algorithms. The database can be used to study genes expressed in particular stages, tissues, and patterns of interest, and to identify conserved promoter sequence motifs that may play a role in the regulation of such expression. The database is accessible from the address .

**Conclusion:**

By combining gene expression, function, and sequence data with integrated sequence comparison algorithms, angaGEDUCI streamlines spatial and temporal pattern-finding and produces a straightforward means of developing predictions and designing experiments to assess how gene expression may be controlled at the molecular level.

## Background

The sequenced genome of the principal vector of human malaria parasites in subSaharan Africa, *Anopheles gambiae *[1], has raised expectations for the development of new and unexpected ways to manage or manipulate vector populations to control disease transmission [2]. As part of efforts to meet these expectations, we generated and organized large data sets using gene expression microarrays to quantify genome-wide transcription in different developmental stages and tissues of this mosquito [3,4]. Arrangement of these data into a searchable format has streamlined the elucidation of genes expressed with stage-, tissue-, and sex-specificity. In addition, by juxtaposing these microarray findings with DNA comparative algorithms, the regulation of genes co-ordinately expressed in specific spatial and temporal patterns can be studied at a mechanistic level. We provide here a public database and web-based data-mining tool that combine stage and tissue expression microarray data, functional annotation, and regulatory DNA sequence comparison algorithms to provide insight into gene expression and regulation in *An. gambiae*.

## Construction and content

### Data collection

Stage-specific transcriptional signal values were imported from genome-wide microarray analyses of *An. gambiae *larvae, male sugar-fed adults, female sugar-fed adults, and female blood-fed adults 3, 24, 48, 72, 96 hours and 15 days after a bloodmeal using Affymetrix GCOS software. Values from tissue-specific microarray analyses also were imported using GCOS to quantify genome-wide transcription in fat bodies, midgut, and ovaries at 24 hours after bloodfeeding [3,4]. Functional gene annotation was imported from the Ano-Xcel database [5] to populate angaGEDUCI with keywords and annotation from the ENSEMBL, NCBI non-redundant, GO, PFAM, and SMART databases. Promoter sequences were selected as regions 1.5 kilobases (kb) in length adjacent to the 5'-ends of transcription start sites of genes using genomic data from ENSEMBL (Assembly: AgamP3, Feb 2006; Genebuild: VectorBase, Feb 2006; Database version: 37.3). Transcription factor binding sites from several classes of organisms were imported from the Transcription Factors Database (TFD) available publicly at . Of the 7,066 sites listed in TFD, 6639 (94.0%) are eight nucleotides or longer and 623 (8.82%) contain degenerate notation. Five-hundred and eleven sites in the database were identified in insects (7.23%), of which 499 (97.7%) are eight nucleotides or longer, and 34 (6.65%) contain degeneracy.

### Implementation

**Figure 1:**
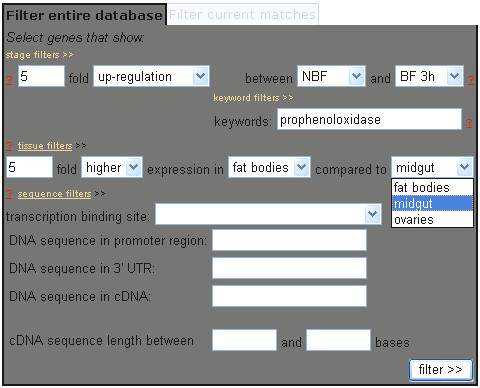
**Data-mining interface**. The "Filter database" data-mining interface allows users to select a gene set that meets specific expression, keyword, and sequence criteria. Input fields include a) differential expression quantified from stage- and tissue-specific expression microarray analyses, b) keywords included in functional annotation gathered by Ano-Xcel [5] from the ENSEMBL, NCBI non-redundant, PFAM, GO, and SMART databases, and c) presence of transcription factor binding sites and other conserved DNA sequences contained within promoter, 3' UTR, or coding regions of the *An. gambiae *genome. Each filter is imposed on the current gene set being examined, beginning with the entire *An. gambiae *genome, thus selecting and reducing the gene set in a stepwise fashion as genes matching previous filter criteria are eliminated by subsequent filters. The parameters specified here are those that are used in the prophenoloxidase case study described in the text.

The data have been stored as a MySQL relational database that is accessible directly through an Apache web server. A web-based data mining interface is used to manage queries to identify genes that meet specific expression, keyword, and sequence criteria (Figure 1). A sequence comparison program based on the Boyer-Moore algorithm [6] is built into the data-mining interface for comparison of promoter regions of genes within a selected gene set.

### Data retrieval

**Figure 2:**
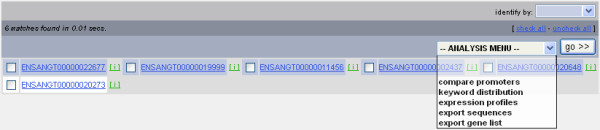
**Gene set with analysis menu**. The six transcripts comprising the prophenoloxidase case study gene set, listed by ENSANGT identifiers, are shown in the background. The link to each transcript invokes a gene entry page, an example of which is represented in Figure 3. The analysis drop-down menu allows users to execute a search for conserved DNA sequence motifs in the promoter regions of the six genes in this gene set, build a keyword distribution from the functional annotation of these genes, display expression profiles of genes in the set, export promoter, 3' UTR, or cDNA sequences of the genes in FASTA format, or export the gene set.

The main page of the database provides hyperlinks to: Filter Database, Import Gene Set, Download Data, View Database, Submit Study, Documentation, and Contact. Selection of the Filter Database link opens the data-mining interface and allows users to focus on specific genes that satisfy input criteria based on: 1) stage- and tissue-specific expression, 2) annotated keywords, 3) DNA sequences present in promoter, 3' untranslated regions (UTR), or coding regions, or 4) presence of specific transcription factor binding sites (Figure 1). Queries are conducted by stepwise entry of input criteria with each query imposed on the previous so that all genes currently displayed meet all preceding query criteria as well as the criterion that was last entered. Once a gene set of interest has been selected, users then can use the analysis menu in the interface to search for conserved DNA motifs within the promoters of the gene set, view expression profiles, build a distribution of annotated keywords, or export the set for future retrieval (Figure 2). Detailed annotation and expression data for each gene also can be viewed at any time by selecting the gene identifier link to invoke the description of a gene entry.

### Description of a gene entry

**Figure 3:**
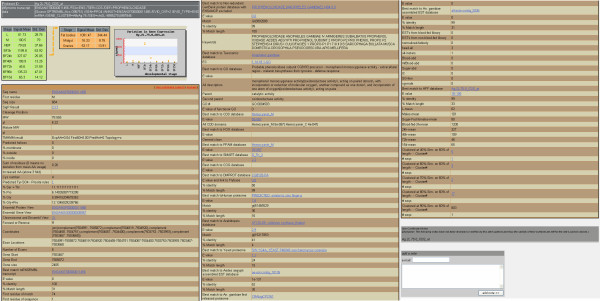
**Gene entry for one transcript**. Complete gene description for one transcript, ENSANGT00000011456. Each entry displays the developmental expression profile built for the transcript from stage- and tissue-specific microarray analyses, followed by a link to Vectorbase and functional annotation gathered by Ano-Xcel [5] from the ENSEMBL, NCBI non-redundant, PFAM, GO, and SMART databases. The bottom of each entry includes user-contributed notes if they are available, as well as a form for users to submit their own notes for immediate listing.

Each gene has a corresponding data page that can be accessed by selecting the gene identifier link during data retrieval. Gene entry pages display data from microarray expression analyses for stage- and tissue-specific expression and functional annotation as gathered by Ano-Xcel from ENSEMBL, NCBI non-redundant, GO, PFAM, and SMART databases (Figure 3). A link to the Vectorbase database that contains additional, centralized gene data also is provided on each entry page. User-contributed notes and a form for sharing notes for a gene entry are found below the annotation of each gene. To encourage data sharing, note submission does not require user pre-registration.

### Comparing promoters to identify conserved DNA sequence motifs

After clustering genes into gene sets that show similar patterns of expression, the data-mining interface analysis menu can be used to search for common DNA motifs that may act as regulatory sequences in coordinating these expression patterns. Two parameters must be selected to begin the analysis: 1) motif match length: the desired conserved sequence motif length to search for in the analysis, 2) mismatches: the number of base mismatches allowed between two nearly-conserved sequence motifs without disqualification.

**Figure 4:**
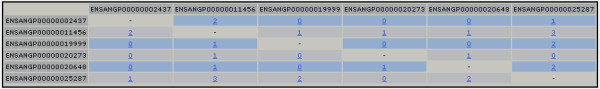
**Promoter comparison matrix**. Each transcript in the current gene set is displayed in a matrix indicating the number of conserved motifs found between each transcript when compared pair-wise with every other transcript within the gene set. The matrix shown corresponds to the prophenoloxidase case study gene set, with the promoter regions of the six transcripts being compared to search for conserved DNA sequence motifs that are 12 nucleotides in length, with no mismatched bases allowed. Each link in the matrix invokes the sequence comparison output shown in Figure 5.

**Figure 5:**
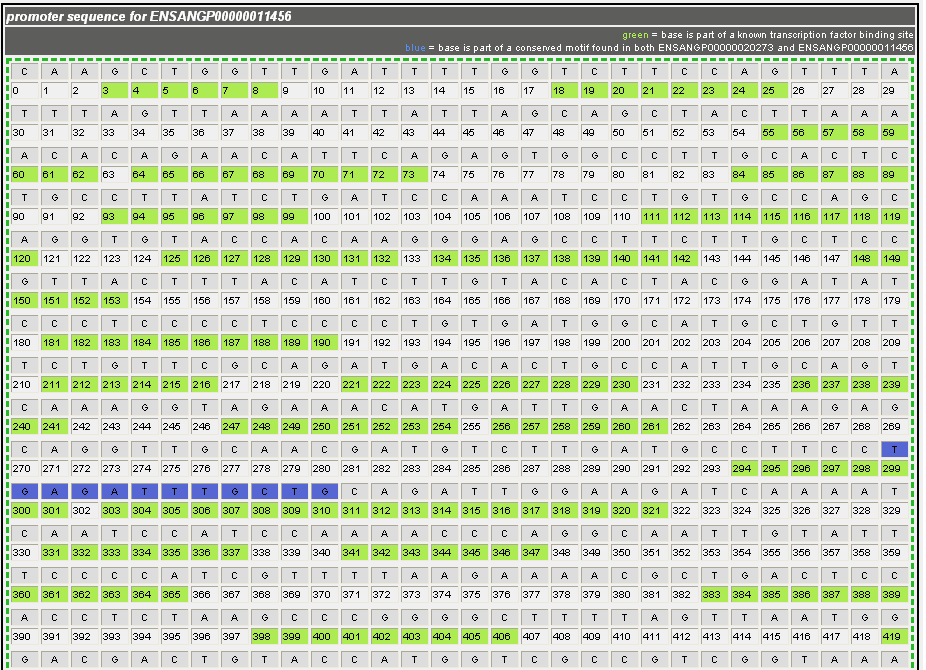
**Promoter sequence comparison between the genes encoding two transcripts**. Abbreviated promoter region for the gene corresponding to one transcript, ENSANGP00000011456 as printed when compared to a second, ENSANGP00000020273. Nucleotides that are part of a conserved DNA sequence motif (of length greater than or equal to the specified motif search length: 12 bp in this example) that is found in both transcripts are indicated in blue. Numbered positions where known transcription factor binding sites occur are highlighted in green.

**Figure 6:**
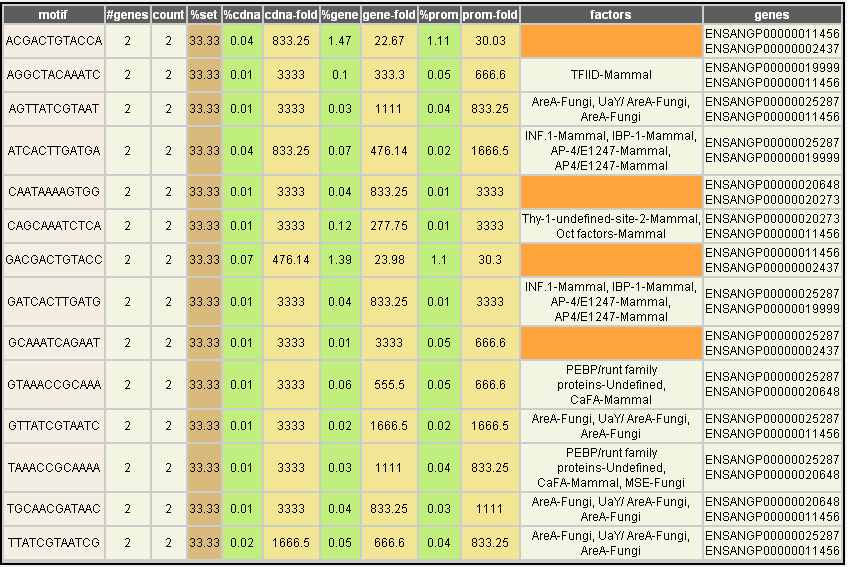
**Conserved DNA sequence motifs in putative promoter regions**. Analysis output from comparing putative promoter regions of the six prophenoloxidase transcripts identified in the case study, searching for conserved DNA sequence motifs that are 12 nucleotides in length with no mismatches allowed. Each conserved DNA sequence (**motif**) is followed by the number of genes (**#genes**) within the gene set where this motif was found, the total occurrences of the motif (**count**), taking into account that some genes may contain multiple instances of a motif, the corresponding frequency (**%set**) of occurrence of this motif within the current gene set, the frequency of occurrence of the motif within: all cDNAs (**%cdna**), all genes [including introns] (**%gene**), and all promoters (**%prom**), in the *An. gambiae *genome, and the fold difference between the frequency of occurrence of the motif in this gene set as compared to its frequency in all cDNAs (**cdna-fold**), all genes (**gene-fold**), and all promoter regions (**prom-fold**), in the *An. gambiae *genome. Each transcription factor binding site that matches or occurs within a conserved motif is indicated (**factors**), along with the class of organism in which the binding site was described originally. Motifs that do not match or contain a known transcription factor binding site are highlighted in orange. The gene identifiers containing each sequence motif are shown in the last column (**genes**).

**Figure 7:**
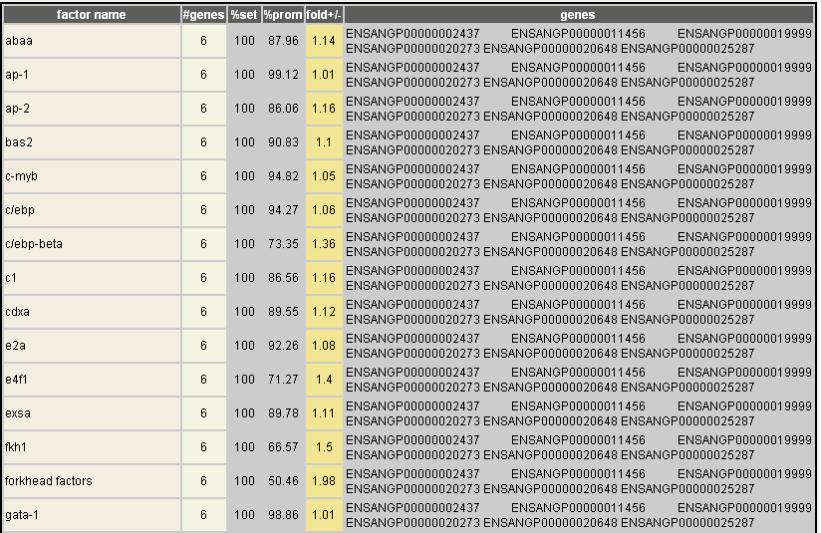
**Transcription factor binding sites contained in a gene set**. Tabular account of known transcription factor binding sites of any length found within the putative promoter regions of the prophenoloxidase case study gene set. Each factor is indicated (**factor name**), along with the number of genes in which it is found (**#genes**), its frequency (**%set**) within the current gene set as compared to its frequency (**%prom**) within all promoter regions in the *An. gambiae *genome, and the difference between the latter two (**fold+/-**). The transcript identifiers containing each transcription factor binding site are indicated last (**genes**). Fifteen of the 287 binding sites found in the case study comparison are shown in this abbreviated figure.

The resulting output from the analysis contains three parts. First, a comparison matrix is displayed indicating the number of conserved motifs found in each pair-wise comparison among every gene in the gene set (Figure 4). Each link in the matrix invokes a new page that prints the promoter sequences of the two genes being compared with areas of sequence conservation and transcription factor binding sites highlighted (Figure 5). Second, a table of the conserved motifs is displayed that compares the frequency of occurrence of each conserved motif within the gene set against the frequency of each motif in all 1) exons, 2) exons and introns, and 3) promoters within the *An. gambiae *genome (Figure 6). Each motif that matches or contains a transcription factor binding site is indicated in the same output. The third item displayed is a table indicating the frequency of occurrence of each transcription factor binding site of any size found within the gene set (Figure 7). Due to the degeneracy and varied size of transcription factor binding sites in the TFD database, the frequencies reported here are noticeably higher in this item compared to the frequencies in the conserved motif table that precedes it.

### Visualization of transcription profiles

**Figure 8:**
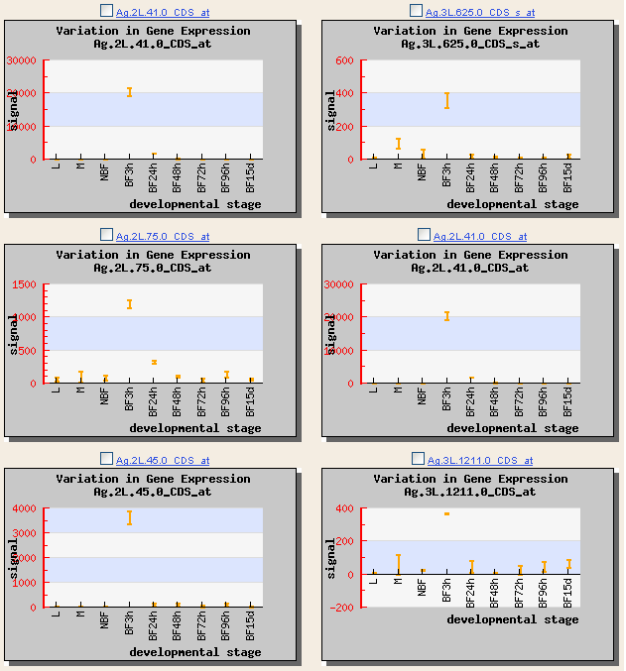
**Developmental expression profiles**. Gene expression profiles measuring transcriptional signal values from stage-specific microarray analyses of the six prophenoloxidase case study transcripts. The stages shown are larvae (L), male (M), sugar-fed adult female (NBF), and blood-fed adult female 3, 24, 48, 72, 96 hours, and 15 days after bloodmeal (BF3h-BF96h, BF15d).

The transcription profiles for a gene set can be viewed in batch by using the analysis menu from the data-mining interface after a gene set has been selected. The resulting graphs print transcriptional expression according to developmental stage: larvae, male sugar-fed adults, female sugar-fed adults, and female blood-fed adults 3, 24, 48, 72, 96 hours and 15 days after a bloodmeal (Figure 8).

### Keyword distribution

**Figure 9:**
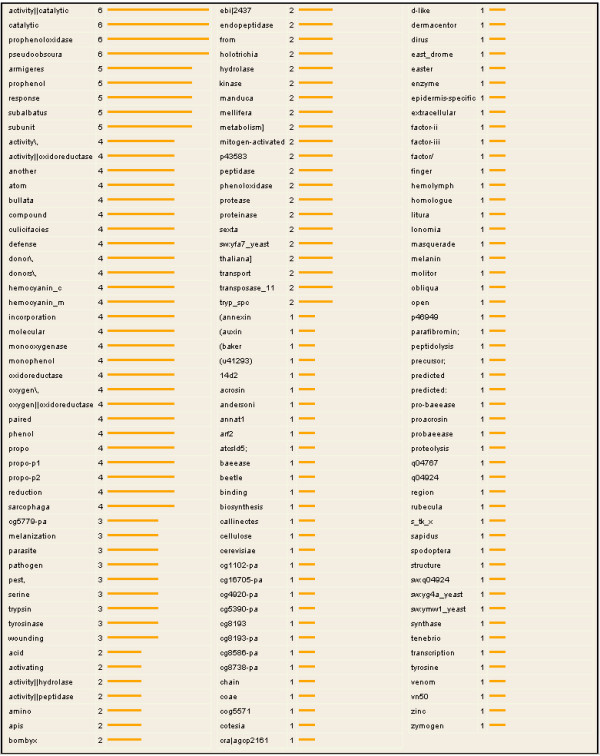
**Keyword distribution**. A distribution of keywords gathered by Ano-Xcel [5] from the ENSEMBL, NCBI non-redundant, PFAM, GO, and SMART databases for genes in the prophenoloxidase case study gene set. The number of occurrences corresponds to the number of genes in the gene set that contain the keyword.

A keyword distribution listing all keywords found in a gene set, as gathered by Ano-Xcel [5], and their respective frequency of occurrence, can be constructed by using the analysis menu from the data-mining interface (Figure 9).

### Import gene set

**Figure 10:**
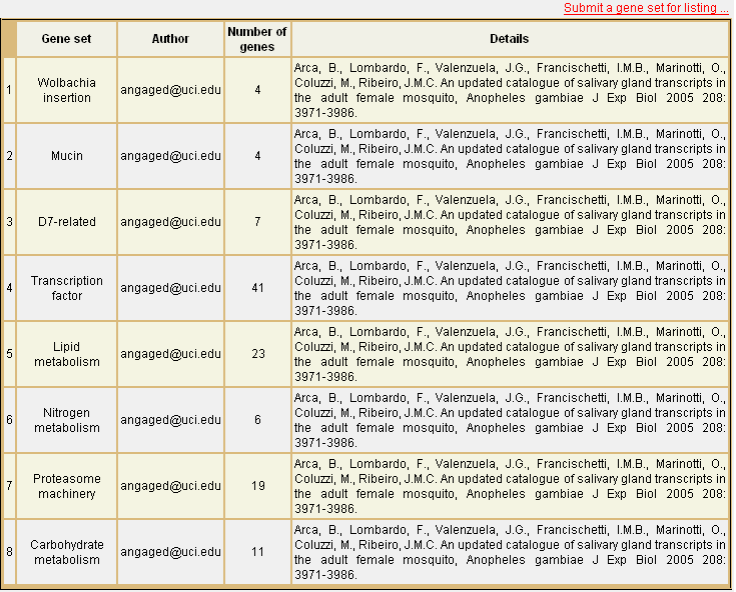
**Pre-defined gene sets**. The "Import Gene Set" page contains a sample list of pre-defined gene sets as grouped in existing literature. Investigators can use the same page to load a pre-defined set into the data-mining interface for study, or to submit additional sets for immediate listing. A general name is provided for each set (**Gene set**) along with the name or e-mail address of the user who submitted the set (**Author**), the number of genes contained in the set (**Number of genes**), and any details about the set or the literature it was derived from (**Details**).

A gene set can be imported by entering a list of gene identifiers in ENSANGG, ENSANGP, ENSANGT, Probeset ID, or Celera form, or by choosing from a list of pre-defined gene sets. Pre-defined gene sets consist of groups of genes that have been linked to similar function or regulation in existing literature (Figure 10). Users can submit gene sets for automatic and immediate listing as a pre-defined gene set from the same page. Gene sets can be exported from the data-mining interface by using the analysis menu.

### Submit a microarray study

The angaGEDUCI database has the capacity to store and integrate additional Affymetrix microarray studies that examine gene expression in *An. gambiae*. The Submit Study link provides a short form for uploading microarray data and specifications.

## Utility and Discussion

The angaGEDUCI database identifies genes that meet stage- and tissue-specific expression criteria, and incorporates keyword searching and promoter sequence analysis into one unified data-mining tool. A case study best illustrates the utility of this integration. In this example, we will identify genes linked to the complex regulation of phenoloxidase, an enzyme involved in the melanization of invading parasites and micro-organisms as part of invertebrate innate immunity [7,8]. Specifically, we will search for pro-phenoloxidase genes that are preferentially found in fat bodies and expressed highly three hours after bloodfeeding. Three filters will be used to complete this inquiry (Figure 1). First, a filter selects genes that contain the keyword "prophenoloxidase" in their functional annotation. Eighty-eight of the 13,639 transcripts in the *An. gambiae *genome contain this keyword. Second, a stage-specific filter identifies 14 of these 88 transcripts that show 5-fold up-regulated expression three hours after bloodfeeding (BF3h) as compared to sugarfed mosquitoes (NBF). Third, a tissue-specific filter isolates six of these 14 transcripts that are expressed 5-fold higher in fat bodies as compared to their corresponding expression in the midgut and ovaries (Figure 2).

**Figure 11:**
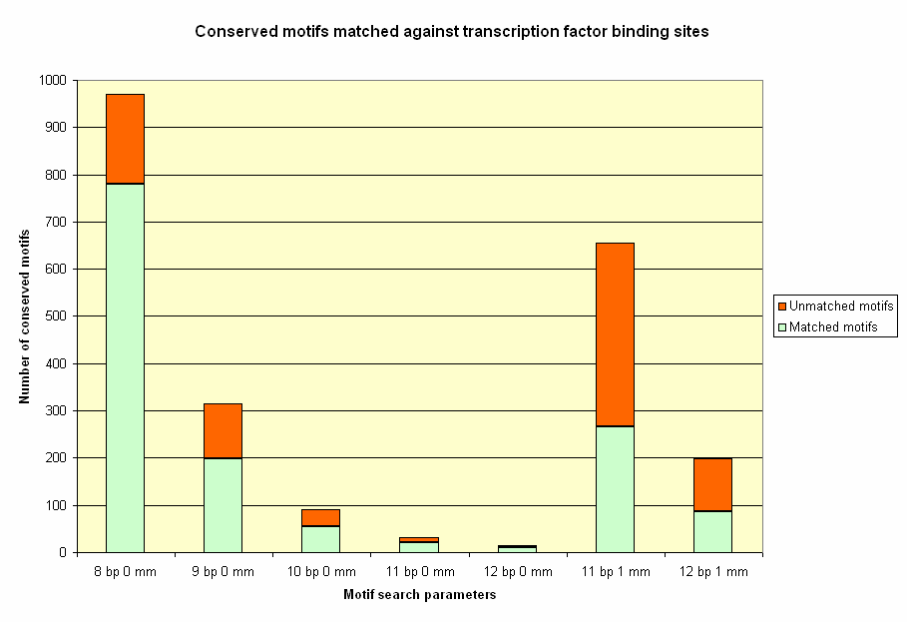
**Promoter analysis results with different parameter specifications**. Different numbers of conserved DNA sequence motifs found by the promoter analysis algorithm when different parameters were specified (x-axis: length in basepairs [**bp**]; number of mismatches allowed [**mm**]). Numbers of conserved motifs (Y-axis) that match known transcription factor binding sites are shown in green, with motifs that do not match known sites shown in orange.

The analysis menu can be used with this gene set of interest to search for common DNA sequence motifs that occur within the promoter regions of the genes corresponding to these transcripts. Analysis of the promoter regions of the six prophenoloxidase-related genes shows the occurrence of 14 conserved 12-basepair DNA sequence motifs (Figure 6). Of these 14 motifs, 10 match known transcription factor binding sites while the other four do not. Additional motifs of interest can be found by executing the promoter analysis as a search for a conserved motif length less than 12 nucleotides in length or by specifying a number of mismatches that may be allowed within a nearly-conserved but imperfectly-matching motif. Depending on how these parameters are adjusted, the output from the promoter analysis of a gene set may generate more or less conserved motifs, as well as a different number of motifs that are or are not matched to known transcription factor binding sites. A survey of the data produced with different specifications of these parameters in the analysis of the prophenoloxidase gene set is included in Figure 11 to aid users in choosing parameters that are most appropriate for their particular investigation.

## Conclusion

While existing databases may allow individualized searching by expression, keyword, or sequence criteria, it is the unification of these fields that makes angaGEDUCI a unique facilitator of experimental design. The database may be used in many different ways, but perhaps most useful is the ability to use the stage- and tissue-specific expression microarray data to identify genes that are expressed in spatial and temporal patterns of interest and then compare the promoter regions of such genes to investigate putative means of facilitating such expression. The experimentally validated utility of such applications may pave the way for similar investigations into the regulatory role of conserved DNA sequence motifs in other control regions within the genome, such as putative microRNA target sites that may be found in 3' UTRs.

In addition to its current microarray data based on genome-wide tissue- and stage-specific gene expression, angaGEDUCI has been built with the goal of expanding its scope to house, integrate, and display additional microarray studies of *An. gambiae*. For example, Affymetrix microarray data from a study investigating gene expression in *An. gambiae *following infection with *Plasmodium falciparum *can be integrated with the existing data in the database to produce a clearer picture of how the mosquito responds to parasite challenge at the transcriptional level. This flexibility assures that angaGEDUCI is capable of growing alongside the increasing quantity of data being produced from other studies. By working closely with Vectorbase and other laboratories in this way, it is hoped that angaGEDUCI will act as a catalyst in accelerating the study and understanding of gene expression and regulation in this important and devastating vector of disease.

## Availability and requirements

The *Anopheles gambiae *Gene Expression Database at UCI is publicly accessible from the URL: . Questions and comments are welcomed through the site.

## Authors' contributions

SND designed and implemented the website, database, and promoter analysis algorithms and wrote the principal draft of the manuscript. OM assisted in designing the analysis and editing of the manuscript. JMCR captured putative promoter sequences and constructed the Ano-Xcel database. AAJ assisted in the editing of the manuscript.
